# Identifying optimal tumor-associated antigen combinations with single-cell genomics to enable multi-targeting therapies

**DOI:** 10.3389/fimmu.2024.1492782

**Published:** 2024-11-07

**Authors:** Matthew A. Nix, Caleb A. Lareau, Jeffrey Verboon, David G. Kugler

**Affiliations:** ^1^ Cartography Biosciences, South San Francisco, CA, United States; ^2^ Department of Laboratory Medicine, University of California, San Francisco, San Francisco, CA, United States; ^3^ Memorial Sloan Kettering Cancer Center, New York, NY, United States

**Keywords:** antibody, multi-specifics, bispecifics, ADC, cell engager, single-cell sequencing, immunotherapy

## Abstract

Targeted antibody-based therapy for oncology represents a highly efficacious approach that has demonstrated robust responses against single tumor-associated antigen (TAA) targets. However, tumor heterogeneity presents a major obstacle for targeting most solid tumors due to a lack of single targets that possess the right on-tumor/off-tumor expression profile required for adequate therapeutic index. Multi-targeting antibodies that engage two TAAs simultaneously may address this challenge through Boolean logic-gating function by improving both therapeutic specificity and efficacy. In addition to the complex engineering of multi-targeting antibodies for ideal logic-gate function, selecting optimal TAA combinations *ab initio* is the critical step to initiate preclinical development but remains largely unexplored with modern data-generation platforms. Here, we propose that single-cell atlases of both primary tumor and normal tissues are uniquely positioned to unveil optimal target combinations for multi-targeting antibody therapeutics. We review the most recent progress in multi-targeting antibody clinical development, as well as the designs of current TAA combinations currently exploited. Ultimately, we describe how multi-targeting antibodies tuned to target pairs nominated through a data-driven process are poised to revolutionize therapeutic safety and efficacy, particularly for difficult-to-treat solid tumors.

## Clinical observations from mono-targeting therapies motivate multi-specifics

The inception of antibody-based therapy four decades ago ushered in a new era of powerful targeted treatments for multiple cancers. While most are canonical IgGs, a smaller yet growing number are more complex antibodies that simultaneously engage two or more targets. The therapeutic effector modalities employed for these drugs span heterogenous formats: monoclonals that elicit ADCC responses, antibody-drug conjugates (ADCs) that deliver cytotoxic payloads, and more recently bispecific cell engagers that harness the potent cytotoxicity of the immune system. Though the number of antibodies and their methods of delivering effector function vary, nearly all approved oncology-focused antibodies engage tumors via binding to a single tumor-associated antigen (TAA), termed mono-targeting antibodies.

The success of mono-targeting antibodies has been driven by rational selection of cancer cell TAAs with considerations for balancing safety, efficacy, and toxicity. Many of the first successful antibodies target single TAAs for the treatment of blood cancers, including CD19 for B-acute lymphoblastic leukemia (B-ALL), CD20 for Non-Hodgkin’s Lymphoma (NHL), and BCMA for multiple myeloma (MM), all of which are expressed exclusively in the hematopoietic system (1–3). As these antigens are not expressed outside of B-cell lineages, off-tumor toxicity is manageable. However, we emphasize that B-cell aplasia is observed in recipient patients and attributable to on-target, off-tumor killing of healthy B-cells. Regardless, the therapeutic targeting of these TAAs demonstrate impressive clinical results, owed largely to the sufficient therapeutic index these targets afford.

Though blood cancer treatment has been revolutionized by mono-targeting therapies, analogous options for solid tumor indications have found less clinical impact. Tumor heterogeneity and TAA expression in critical healthy tissues result in a narrow therapeutic window, requiring extensive considerations for both TAA targeting and biologic engineering. Despite challenges implicit to targeting solid tumors, certain approved antibody therapies targeting individual TAAs have demonstrated robust clinical benefit. One of the most notable examples is Trastuzumab (targeting HER2) which conveys significant improvement to overall survival for breast cancer patients (4). ADCs targeting individual TAAs including HER2, CD142 (tissue factor), FR alpha, Nectin-4, or Trop-2 display robust responses against various solid tumor indications, resulting in multiple FDA approvals for this class of drugs and stimulating wide preclinical investment (5). Furthermore, several cell engagers directed at TAAs in solid tumors have shown clear clinical benefit and resulted in FDA approval, including Tarlatamab (targeting DLL3) in small cell lung cancer (6). Broadly, for both blood and solid tumors, there are hundreds of mono-targeting antibody therapies in preclinical and clinical development, including conventional IgGs, ADCs, and cell engagers (7).

Though certain mono-targeting therapeutics have demonstrated real clinical benefit, clear challenges remain that likely limit their efficacy. In most solid tumors, individual TAA expression often extends beyond the primary tumor site to various normal tissue compartments, thereby narrowing the potential therapeutic window. Recent late-stage clinical failures of CEACAM5 mono-targeting therapeutics including the T-cell engager Cibisatamab and the ADC Tusamitamab ravtansine, were likely driven in part by high CEACAM5 target expression in normal GI tissues relative to tumor, resulting in toxicities that prevent achievement of clinically meaningful doses (8, 9). Such toxicities are not unique to CEACAM5 but observed across many other nominated individual targets, including EGFR and MSLN-targeted therapies among others (10, 11).

The limited therapeutic window of existing TAAs motivates the exploration of new targets for future precision therapies. In this regard, multi-targeting antibodies that engage multiple TAAs have emerged as a promising next-generation strategy (12, 13). In this paradigm, multi-targeting AND-gate antibodies require expression of both targets on the same cell for binding and effector function. Ideally, all cancer cells highly express both TAAs whereas normal tissues express zero or only one TAA at physiologic levels. Thus, successful engineering of each TAA binder to optimize combined avidity enables selective binding to double positive tumor cells. Consequently, single positive only healthy tissues are spared by insufficient binding from either single arm individually (14). Beyond improved discrimination of tumors versus normal tissue, multi-targeting antibodies also function via different mechanisms depending on target pair and modality choice. Although clinical data has yet to emerge for most multi-targeting antibodies, early preclinical research has begun to define general principles for multi-targeted antibody design, including binder format, architecture, affinity, and avidity to anticipate the functional response of these therapies (15).

To date, most efforts to develop multi-targeting biologics have focused on enacting advanced antibody design principles. In addition, antigen pairs targeted by these solid tumor therapies have focused narrowly on a small set of well-explored TAAs (e.g. EGFR, cMET, etc). Target pair selection has typically relied upon sub-optimal methods of TAA characterization, including bulk RNA expression analysis or low-resolution immunohistochemical staining (IHC). However, these approaches are poorly suited to define single or co-expression of TAAs in tumor or normal tissues at the resolution required for optimal therapeutic targeting. We hypothesize that overall clinical success of multi-targeting antibodies will rely not only on sophisticated antibody engineering, but also on rigorous selection of appropriate target pairs. Here, we describe how single-cell atlases of both primary tumor and normal tissues with their exceptional high-resolution vantage point of target pair expression can fulfill this function. We highlight recent examples of solid tumor multi-targeting antibodies in clinical development that target previously established TAAs identified through conventional technologies. We describe how multi-targeting antibodies that are directed towards target pairs nominated through an unbiased, data-driven process promise to dramatically improve overall patient outcomes for difficult-to-treat solid tumors.

## Multi-targeting antibodies, ADCs, and cell engagers as emerging therapies

**Figure 1:**
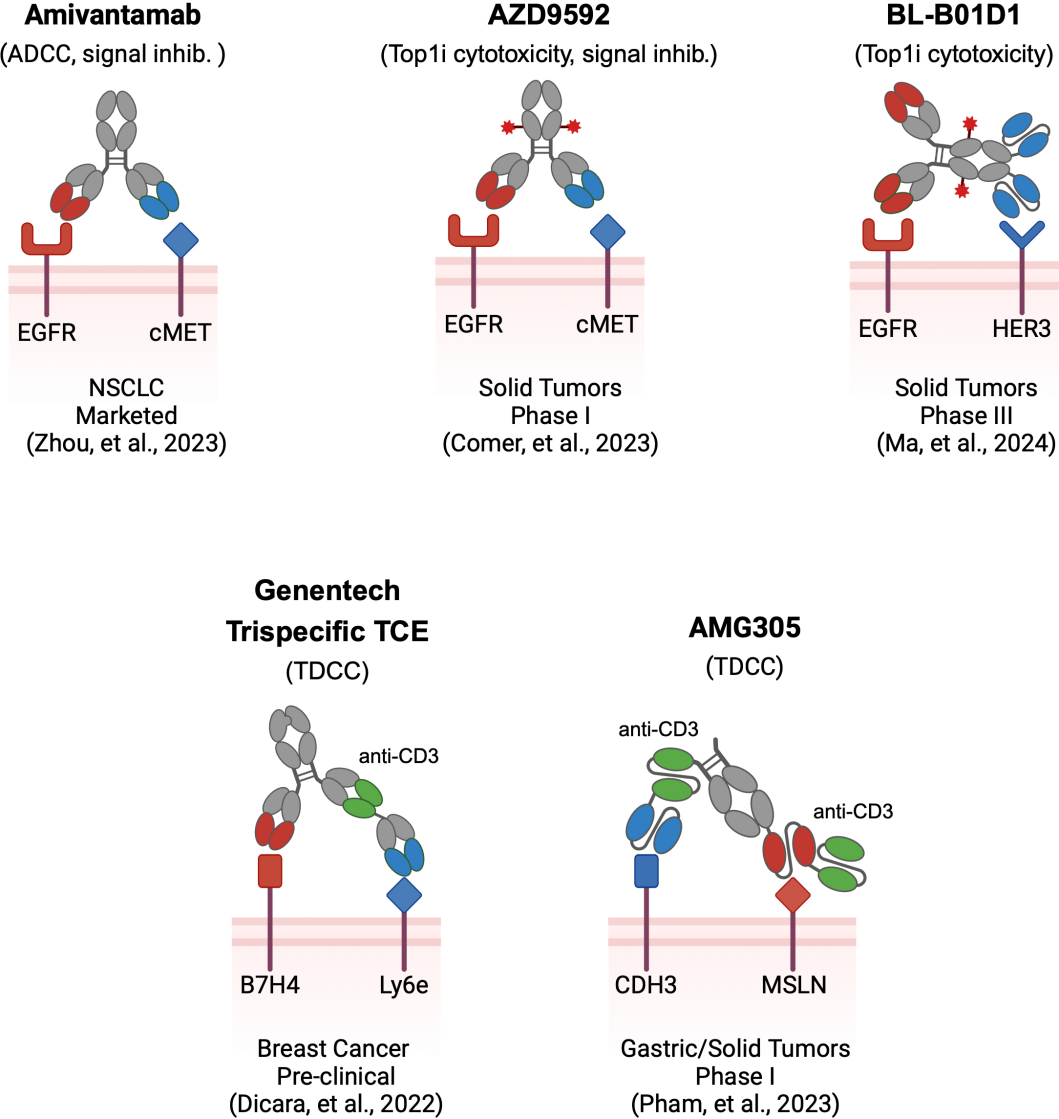
Examples of multi-targeting antibody therapeutics. Multi-targeting antibodies utilize different architectures and effector modalities, in addition to the antigens they target. Several pioneering multi-specific biologics are shown in schema form. Amivantamab is a bispecific antibody utilizing two Fab binders against EGFR and cMET with ADCC and signal blocking effector function. AZD9592 is a bispecific ADC with two Fab binders against EGFR and cMET that delivers a TOP1i drug payload. BL-B01D1 is a tetravalent bispecific ADC with Fab and scFv binders against EGFR and HER3, delivering a TOP1i drug payload. Genentech’s B7H4-Ly6e trispecific T-cell engager utilizes three Fab binders assembled using CrossMab technology for targeting Breast Cancer. AMG305 is a CDH3-MSLN directed, trispecific Fc-containing BiTe, utilizing three scFv binders for treating various solid tumors. Binders are color-coded to reflect the TAA they target. Red stars depict ADC payload conjugates.

Though multi-targeting antibody therapeutics are in their infancy, the principles behind this approach have been explored utilizing multiple modality formats. The most clinically advanced example of a multi-targeting antibody is the FDA-approved bispecific Amivantamab, which simultaneously targets EGFR and cMET for the treatment of non-small cell lung cancer (**Figure 1**). Amivantamab exerts tumor inhibition via heterogenous mechanisms resulting from dual-TAA engagement, including inhibition of EGFR-cMET receptor activation, receptor degradation, and Fc-effector mediated ADCC killing (16). Reduced skin toxicity in non-human primate (NHP) models relative to mono-targeting antibodies suggests enhanced tumor selectivity with bispecific avidity likely playing a major role. Double-positive EGFR-cMET tumors are preferentially targeted while EGFR- only positive skin tissues are spared due to weak monovalent engagement. As the first successful multi-targeting antibody, lessons from Amivantamab have fundamentally shaped this burgeoning field, including the emergent properties of dual-TAA engagement on tumors that incur efficacy via multiple modes of action.

ADCs are composed of chemotherapeutic agents tethered to antibodies through chemical linkers. Delivery of the drug payload to tumors through target binding, internalization, and trafficking to lysosomes drives potent cytotoxicity that expands the therapeutic window beyond free chemotherapeutic agent. Analogous to the principles of unconjugated bispecifics like Amivantamab, multi-targeting ADCs hold potential to address several key limitations of mono-targeting ADCs (12). Specifically, multi-targeting ADCs can be tuned to favor avid binding to dual-positive tumors to achieve AND-gated selectivity, delivering cytotoxic payloads specifically to tumor cells. As an additional hurdle many individual TAAs exhibit poor internalization kinetics resulting in diminished drug delivery to tumors. Conversely, multi-targeting ADCs may enhance internalization through hetero-receptor crosslinking, driving rapid ADC endocytosis that translates to superior drug delivery. Several multi-targeting ADCs are currently being evaluated in clinical trials targeting a narrow selection of TAAs including EGFR, cMET, MUC1, and HER3. AZD9592, as one example is a bispecific ADC targeting EGFR and cMET for delivery of a topoisomerase I inhibitor currently in phase I trials (17) (**Figure 1**). In preclinical evaluation, AZD9592 displayed potent cytotoxicity for EGFR-cMET double-positive tumors. Single-positive cells displayed diminished susceptibility, confirming enhanced selectivity and AND-gate function. Internalization was also enhanced through simultaneous engagement of EGFR and cMET whereas pre-blocking either target with monoclonal antibodies reduced internalization, confirming that receptor crosslinking enhances ADC endocytosis. Buoyed by this rationale for therapeutic activity, several other multi-targeting ADCs are currently being evaluated clinically (18, 19).

Yet another modality is multi-targeting cell engagers that similarly direct cytotoxicity to solid tumor cells marked by multiple TAAs. These biologics are designed with three binder arms against two TAAs as well as CD3 for T-cell effector function (**Figure 1**). The most clinically advanced agent is AMG305, which simultaneously targets the CDH3 and MSLN receptors (20). Via affinity tuning of each TAA binder, AMG305 can target CDH3-MSLN double positive tumor cells while attenuating cytotoxicity of single-target healthy cells. Based on positive preclinical efficacy and safety in NHP studies, AMG305 has recently entered into a phase I clinical trial. Additionally, Dicara et al. developed a tri-specific T-cell engager for breast cancer targeting Ly6e and B7-H4, selected based on re-analysis of historical IHC data (21). The authors explored the impact of binder arrangement as well as CD3 affinity in their trispecific format, demonstrating successful tumor control while also minimizing toxicity *in vivo*.

Taken together, multi-targeting antibodies demonstrate marked improvements over mono-targeting approaches against these same targets in preclinical evaluations. Whether these biologics are developed in bispecific antibody, bispecific ADC, or trispecific cell engager formats requires considerations implicit to each modality though all feature the multi-target-based avidity tuning to achieve specificity. Although not covered here, we note that similar multi-targeting advancements have also been applied to cell therapy modalities both preclinically and clinically (22–24). Enhancements in tumor cell specificity and emergent functional properties unique to each format drive this improvement. Clinical readouts from ongoing multi-specific trials will continue to shape the appropriate modality for future therapeutic development.

## Single-cell genomics for data-driven exploration of combinatorial expression

While careful antibody engineering is required to optimize multi-targeting function, the choice of TAAs is paramount to eventual success of multi-targeting agents, which will ultimately shape the therapeutic index. In particular, the selection of highly potent modalities like cell engagers (and cell therapies) underscores the necessity of critically assessing TAA single and co-expression to avoid unforeseen toxicity (25). Assessing current multi-targeting therapeutics, we observed that most focus on a few well-described TAAs discovered and validated through compendial measures of tissue expression (**Figure 1**).

**Figure 2:**
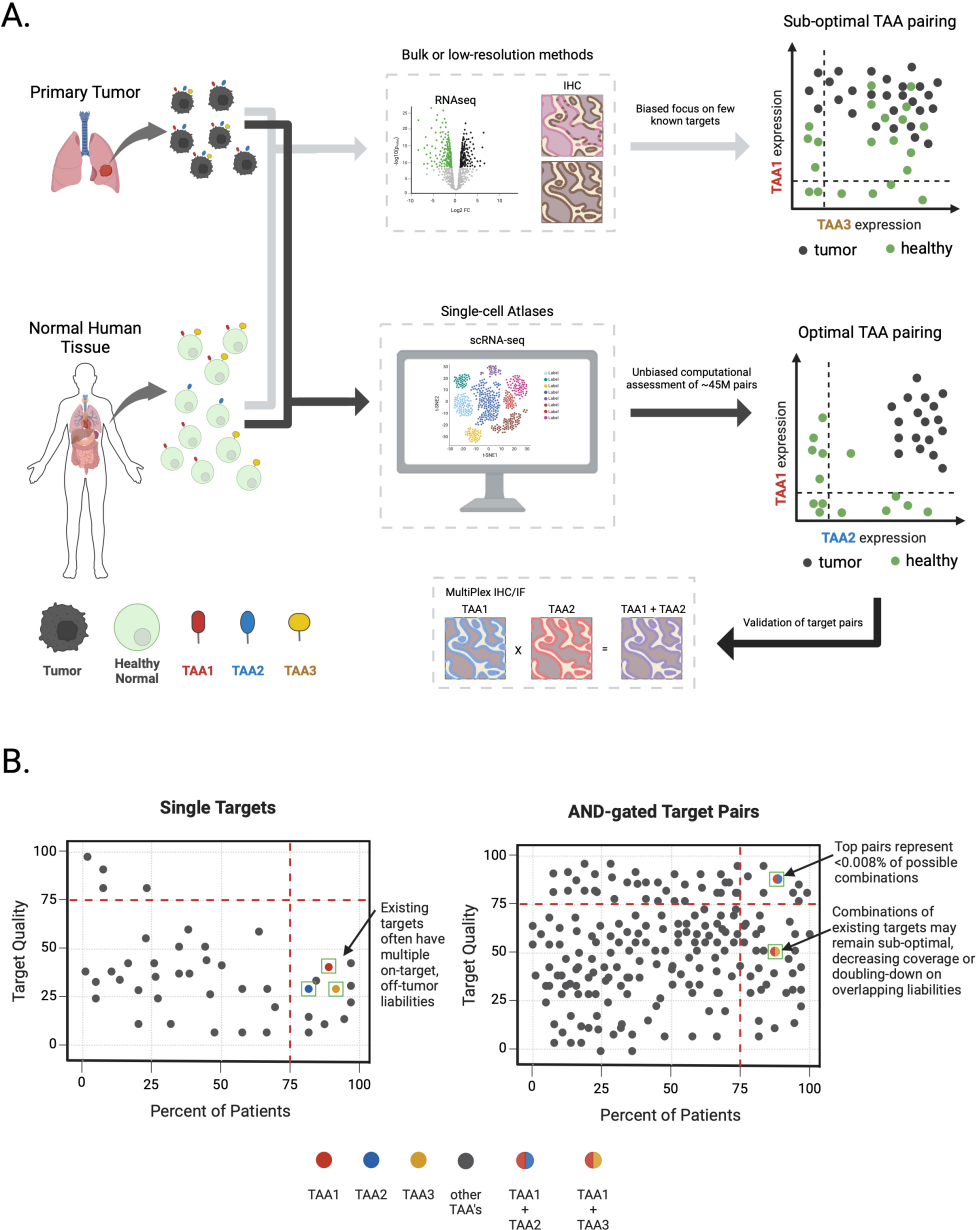
Identifying optimal target pairs for AND-gate therapies via single-cell sequencing atlases. **(A)** Target expression profiling in primary tumor samples or healthy normal tissues through conventional means (e.g.; bulk RNAseq or Immunohistochemistry) obscures true TAA expression patterns and may lead to nomination of sub-optimal TAA pairs for multi-targeting approaches (light grey arrows). Large-scale, single-cell atlases combined with computational assessment of all ~45M possible cell surface TAA combinations enables an unbiased, data-driven approach to measure true TAA expression patterns and nomination of optimal TAA pairs followed by validation at the protein level for AND-gate therapeutics development (black arrows). Tumor and healthy cells are colored black or green, respectively. TAAs and their hypothetical expression profiles on tumor and healthy cells are depicted with color coding (i.e.; red – TAA1, blue – TAA2, yellow – TAA3). Plots depicting true TAA co-expression in tumor or healthy cells are shown on the far right. **(B)** Schematic depicting target quality features for TAAs alone or in combination for AND-gate target pairs, derived from scRNA-seq solid-tumor and healthy tissue atlases. Left graph depicts single TAAs (depicted as circles) plotted according to a relative target quality score (y-axis) versus percent of patients in a disease indication expressing a given TAA (x-axis). Examples of theoretical existing single targets depicted as colored circles (i.e.; red – TAA1, blue – TAA2, yellow – TAA3). Higher target quality scores reflect superior target expression profile in healthy tissues (i.e.; a very clean on-target, off-tumor putative therapeutic index) while higher percent of patients reflects overall TAA expression coverage in tumors. For brevity, only a few TAAs are depicted from ~6,000 cell surface genes. Right-most panel depicts AND-gated TAA combinations graphed on the same axes. Theoretical TAA combinations are depicted as two-color circles where each color represents different TAAs (i.e.; red + blue circle = TAA1 x TAA2, red + yellow circle = TAA1 x TAA3). Only a few TAA combinations are depicted from ~45M potential combinations.

Recognizing that these multi-specific therapeutic agents direct killing on individual cells, there is an obvious need to characterize the co-expression of two TAAs at the same resolution. Bulk measurements such as traditional RNA-seq and IHC do not possess the requisite granularity for accurate TAA pair assessment. Consequently, these traditional approaches combined with the bias towards classically known targets may lead to selecting sub-optimal TAA combinations. Instead, we propose that data-driven single-cell sequencing analyses, integrating both tumor and normal tissue atlases, achieves the quantitative resolution required to nominate optimal TAA combinations (**Figure 2**).

Due to the distinct scalability (~10^5^ cells per library) and broad applicability across tissue and tumor types of single-cell sequencing, TAA co-expression can be readily evaluated in all populations, from individual tumor cells to single cells encompassing normal tissues. Thus, a nearly complete map of TAA expression across cell types throughout the human body can be developed that accounts for not only highly abundant cells but also rare cell populations where TAA expression status may be obfuscated through bulk measurements. We emphasize that rare cell states or minor cellular subpopulations may play an outsized role in determining safe and effective TAA combinations (26, 27).

A major obstacle to realizing single-cell data for target pair nomination is the creation and utilization of the atlas itself. While efforts to build a human cell atlas draft have been ongoing for years, a version encompassing all healthy tissues has remained elusive. Creating massive single-cell atlases of hundreds to thousands of healthy/non-cancerous samples that allow a deep understanding of off-tumor toxicity presents both a technical and financial challenge. One widely adopted approach is the aggregation and curation of single-cell studies published in the last decade, albeit at the tradeoff of complex downstream batch effects. An alternative, but far more costly and labor-intensive approach, is *de novo* construction of an atlas tailored for this specific purpose. Either choice requires careful data quality control and integration of samples to remove batch effects while preserving often subtle biological signals captured in sparse single-cell data. Finally, the cells must be annotated with what they represent, i.e. cell type, cell state, at increasing granularity for preclinical therapeutic interpretation. Such annotations of high-dimensional data require deep technical expertise in appropriate dimension reduction statistical theory and biological expertise to delineate healthy and tumor cell types. In the coming years when such an atlas is defined and publicly available, the result will be a complex molecular catalog containing hundreds to thousands of samples, thousands of cell-types and cell-states, and millions of cells, all of which are assessed across the human transcriptome.

## From single-cell atlases to multi-specific targets

While amassing transcriptomic profiles across tumors and healthy tissues is obligatory to enable target identification, the inherent limitations to scRNA-seq data require additional considerations for downstream inferences. Conceptually, the search space of target pairs scales by the number of potential TAAs squared (~*n* ^2^), often resulting in trillions of sparse single-cell data points (**Figure 2B**). With these data challenges in mind, we highlight three key considerations for navigating this vast search space: 1) condensing the data to the relevant genes for the therapeutic modality; 2) grouping these single cells and choosing appropriate summary statistics; and 3) engineering suitable algorithms to nominate gene pairs that discriminate between healthy and malignant cell types.

The first computational challenge of comparing healthy to malignant cell groups is simplifying the data to a minimally relevant set of genes. Filtering all detected transcripts to those corresponding to the cell surface proteome (estimated from 2,000 - 6,000 genes) reduces complexity but still corresponds to ~10^6^-10^7^ million gene pairs (28). Second, collapsing millions of cells into thousands of cell groups is an essential step with single-cell data to account for sparse expression of TAA pairs within any given individual cell. Upon aggregation, the full range of heterogeneity is obscured and hence requires appropriate summary measures to describe the population behavior. Selection of aggregation metrics including mean expression, percent of expressing cells, and definitions to collapse cells (e.g., by cell type, cell state, etc) are all critical to evaluate target pairs, and emphasizes the importance of quality cell labels. Finally, efficient computational methodologies are required to nominate target pairs from scRNA-seq summary statistics. Due to the inherent complexity of this challenge, a method customized for AND-gate target selection has not yet been described, however, two recent advances provide a critical theoretical foundation for this purpose. The first approach seeks the minimal set of marker genes discriminating specific cell populations; wholly applicable to finding AND-gate logic TAA pairs. Recent work has framed this challenge as a cover-set problem allowing the use of efficient mixed integer linear programming and adjustable cell-group summarization choices to find two gene solutions (29). An alternative approach developed encompasses a two-step machine learning framework with an interpretable random forest first step to select informative genes in classified cell groups, followed by a convolutional neural net to identify AND-, OR-, and NOT-gated logic pairs (30).

Despite these computational advances, both methods rely on early evaluation of single genes that distinguish cell populations or are informative to the malignant versus non-malignant classification problem. However, each method compromises on exploration of combinatorial antigen space out of necessity due to inefficiencies in the computational approach. An optimal biological solution in our view would not only evaluate all candidate surface gene pairs, but also enable the comparison of paired-gene expression on malignant versus healthy cell types to model the potential therapeutic window - conceptually, a shared single metric that describes the behavior of both TAAs while capturing the properties of the intended biologic.

As examples of summary statistics, an AND-gate could be described as the minimum expression of two genes wherein the efficacy and toxicity of the drug would be defined by the lesser expression of the pair. An OR-gate might be described as the sum of gene expression. However, highly simplified summary statistics pose their own challenges as enacting the summary requires careful consideration of what specific cells are considered to represent properties of a larger unit (e.g., cell type). Further, summarizing within cell types still requires additional computation to capture the overall risks (potential on-target, off-tumor expression) and benefits (degree of on-tumor expression). Ultimately, these methods should reflect a “target quality” score for not only single TAAs but also all possible TAA pairings (**Figure 2B**).

We suggest that single targets of all TAA pairings be classified by metrics summarizing tumor and patient coverage, ultimately allowing for the nomination of optimal TAA pairings. While this approach is ideal for pair nomination, validation of TAA expression at the protein level is still required using multiplexed IHC or IF. Simply combining existing targets, while attractive at first glance, will necessarily result in sub-optimal AND-gate pairing, thereby imposing a low ceiling on the potential success of new therapeutics from the outset. By our estimates, computationally nominated AND-gate pairs will yield far more convincing and therapeutically innovative biologics to realize the untapped potential of these exciting modalities. Ultimately, proper clinical read-outs of future and as well as current multi-targeting therapies in development will provide invaluable footholds for improving multi-TAA selection and allow for method development that finds the correct balance of efficient computation and target-pair completeness.

## Conclusions

Though multi-targeting antibodies remain nascent, the unprecedented upside of targeting heterogeneous cell types and tumors has generated tremendous enthusiasm, particularly in settings where prior mono-targeting therapies have failed. While selecting TAAs is a critical consideration for any potential therapy, the unique nature of multi-specific agents multiplies the degree of complexity, requiring the characterization of the co-expression of TAAs rather than their behavior individually. Given this complexity, conventional methodologies such as IHC and bulk RNA-seq for target identification are insufficient, particularly given the necessity of characterizing TAA expression on individual cells. We believe that integrated single-cell atlases of primary tumors and normal tissues provide a feasible framework to map the combinatorial landscape and navigate toward optimal targeted immunotherapies. As single-cell technologies readily quantify patterns of TAA co-expression, this foundational measurement boosts the likelihood of identifying successful TAA pairs while simultaneously anticipating potential off-tumor targeting. Together with advanced design principles for generating optimized multi-targeting antibody therapies, we anticipate that these methodologies will lead to improved targeted therapies and superior outcomes for cancer patients.

## Data Availability

The original contributions presented in the study are included in the article/supplementary material. Further inquiries can be directed to the corresponding author.
